# Alteration in basal and depolarization induced transcriptional network in iPSC derived neurons from Timothy syndrome

**DOI:** 10.1186/s13073-014-0075-5

**Published:** 2014-10-10

**Authors:** Yuan Tian, Irina Voineagu, Sergiu P Paşca, Hyejung Won, Vijayendran Chandran, Steve Horvath, Ricardo E Dolmetsch, Daniel H Geschwind

**Affiliations:** Neurogenetics Program, Department of Neurology, Center for Autism Research and Treatment, Semel Institute, David Geffen School of Medicine, University of California, Los Angeles, CA 90095 USA; Interdepartmental Ph.D. Program in Bioinformatics, University of California, Los Angeles, CA 90095 USA; School of Biotechnology and Biomolecular Sciences, University of New South Wales, Sydney, NSW 2052 Australia; Department of Psychiatry & Behavioral Sciences, Center for Sleep Sciences and Medicine, Stanford University School of Medicine, Stanford, CA 94305 USA; Department of Human Genetics, David Geffen Sch. of Medicine, UCLA, Los Angeles, CA USA; Department of Neurobiology, Stanford University, Stanford, CA 94305-5345 USA; Novartis Institutes for Biomedical Research, Cambridge, MA 02139 USA

## Abstract

**Background:**

Common genetic variation and rare mutations in genes encoding calcium channel subunits have pleiotropic effects on risk for multiple neuropsychiatric disorders, including autism spectrum disorder (ASD) and schizophrenia. To gain further mechanistic insights by extending previous gene expression data, we constructed co-expression networks in Timothy syndrome (TS), a monogenic condition with high penetrance for ASD, caused by mutations in the L-type calcium channel, Ca_v_1.2.

**Methods:**

To identify patient-specific alterations in transcriptome organization, we conducted a genome-wide weighted co-expression network analysis (WGCNA) on neural progenitors and neurons from multiple lines of induced pluripotent stem cells (iPSC) derived from normal and TS (G406R in CACNA1C) individuals. We employed transcription factor binding site enrichment analysis to assess whether TS associated co-expression changes reflect calcium-dependent co-regulation.

**Results:**

We identified reproducible developmental and activity-dependent gene co-expression modules conserved in patient and control cell lines. By comparing cell lines from case and control subjects, we also identified co-expression modules reflecting distinct aspects of TS, including intellectual disability and ASD-related phenotypes. Moreover, by integrating co-expression with transcription factor binding analysis, we showed the TS-associated transcriptional changes were predicted to be co-regulated by calcium-dependent transcriptional regulators, including NFAT, MEF2, CREB, and FOXO, thus providing a mechanism by which altered Ca^2+^ signaling in TS patients leads to the observed molecular dysregulation.

**Conclusions:**

We applied WGCNA to construct co-expression networks related to neural development and depolarization in iPSC-derived neural cells from TS and control individuals for the first time. These analyses illustrate how a systems biology approach based on gene networks can yield insights into the molecular mechanisms of neural development and function, and provide clues as to the functional impact of the downstream effects of Ca^2+^ signaling dysregulation on transcription.

**Electronic supplementary material:**

The online version of this article (doi:10.1186/s13073-014-0075-5) contains supplementary material, which is available to authorized users.

## Background

The L-type calcium channel, Ca_v_1.2, plays a central role in regulating an activity-dependent signaling network that is essential for neuronal function [1-6]. A particularly salient example of a perturbation in Ca_v_1.2 function is Timothy syndrome (TS), a rare genetic disorder caused by dominant mutations in the gene *CACNA1C*, which encodes the α subunit of the voltage-gated calcium channel Ca_v_1.2. TS mutations in this subunit result in a conformational change of Ca_v_1.2, leading to delayed channel inactivation and elevation of intracellular calcium upon depolarization [7-9]. TS patients typically exhibit a spectrum of severe nervous system abnormalities, including autism spectrum disorder (ASD) in up to 80% of the patients [8-10]. Given the increasing appreciation for rare monogenic contributions to ASD [11-13], TS provides a powerful avenue for understanding both basic neurobiological processes and ASD pathophysiology.

Given the pleiotropic manifestation of *CACNA1C* mutations in TS and the recent implication of common variation in *CACNA1C* across multiple neuropsychiatric disorders [14], we reasoned that characterization of the Ca_v_1.2-dependent signaling network in TS would help elucidate its molecular basis and prioritize genes for therapeutic development. Although it has been known that calcium influx triggers massive transcriptional changes by acting through several transcription factors, including calcium response factor (CaRF) [15,16], myocyte enhancer factor-2 (MEF2) [17,18], nuclear factor of activated T-cells (NFAT) [19,20], and cAMP response element-binding proteins (CREB) [21-24], little is known about their downstream targets in human neurons and how these processes are altered in disease states such as TS. Here, we reasoned that identifying alterations in mRNA transcript levels in TS patient-derived cortical progenitors and developing neurons would help clarify, not only how calcium regulates gene expression in TS, but more broadly inform our understanding of the molecular mechanism of ASD.

Previously, we reported that the TS mutation was associated with abnormalities in cortical neurogenesis, activity-dependent dendrite retraction, and an excess production of catecholamines [25,26]. Here, to provide a higher order view of the transcriptional changes caused by the TS mutation in *CACNA1C*, we constructed genome-wide transcriptome networks in control and TS neural progenitors and differentiated neurons at rest and following depolarization. Using Weighted Gene Co-expression Network Analysis (WGCNA) [27,28], we identified gene co-expression modules associated with neural development, as well as depolarization shared across both patient and control lines. By comparing TS and control networks, we identified distinct TS related modules enriched in intellectual disability (ID) genes and ASD susceptibility genes. By further integrating the co-expression network with transcription factor binding analysis, we identified candidate regulators for disease-associated modules, including NFAT [19,20], MEF2 [17,18], CREB [21-24], and forkhead box proteins O (FOXO) [29-32]. Our results provide a functional genomic framework for a calcium-dependent signaling network by highlighting the downstream transcriptional targets of Ca_v_1.2 dysregulation, and yields insights into molecular mechanisms relevant to both TS and ASD.

## Methods

### Expression data set

Expression data were obtained from Paşca *et al*. [25] (GSE25542). As previously described, cortical neural progenitors and neurons were generated from independent differentiation of four control iPSC lines from two normal subjects, three TS iPSC lines from one TS patient, and one human embryonic stem cell line (H9). All three subjects included in this expression projects are females. To obtain activity-dependent gene co-expression networks, neurons were treated with 67 mM KCl or vehicle, and harvested after 9 h. The maturation of the specific neuronal cultures has been assessed with Fluidigm Dynamic Arrays and functional characterizations, including patch-clamp recording and live calcium imaging [25]. The analysis showed that at day 42 of differentiation *in vitro* most of the cells were electrically active, and expressed neuronal markers [25]. Moreover, most of the cells are lower layer cortical neurons, and around 20% are upper layer cortical neurons [25]. In terms of electrophysiological features, there are no significant differences between the TS cells and controls cells with regard to their action potential threshold or amplitude, resting membrane potential, input resistance or capacitance [25]. However, by time-lapse video microscopy assay with calcium indicator Fura-2, Paşca *et al*. showed electrophysiological abnormalities in these patient cells compared to controls, including abnormal calcium currents after depolarization and longer action potentials [25].

Total RNA was extracted using the RNeasy Mini kit (QIAGEN). cDNA labeling and hybridization on Illumina HumanRef-8 v3 Expression BeadChips (Illumina) were performed according to the manufacturer’s protocol. Microarray data were analyzed with custom R scripts calling Bioconductor packages. Outlier arrays were detected based on low inter-sample correlations. Raw expression data were log_2_ transformed, and quantile normalized. Probes were considered robustly expressed if the detection *P* value was <0.05 for at least half of the samples in the data set. Consequently, a total of 13,255 expressed genes from 12 neural progenitor cell lines, 15 neuronal cell lines at rest, and nine KCl-depolarized neurons from cases and controls were used for network analysis.

Reproducibility is often an issue in iPSC studies. Here, although we only have one TS patient, two controls, and an additional control H9 ES cell line, we have five neuronal lines from the TS patient, and multiple lines for each control, both at rest and with K^+^ induced depolarization. Within each cell type, cell lines derived from the same subject clustered more closely together than to the cell lines from different subjects. Particularly, the five TS neuronal lines all tightly clustered together. The average intra-subject variance between lines is low: 0.042, 0.053, 0.058, and 0.066 for the TS patient, H9, and the two controls, respectively. Additionally, the experimental data from Paşca *et al*. showed that these lines generated reliable and reproducible cell types at the genome-wide level [25].

### Weighted Gene Co-expression Network Analysis (WGCNA)

We conducted signed co-expression network analysis using the R WGCNA package [27] as previously described [33-35]. WGCNA is based on topological overlap measurements derived from pairwise correlation-based adjacency values to estimate the neighborhood similarity among genes, followed by hierarchical clustering to identify gene co-expression modules. Instead of focusing on individual genes, WGCNA is highly effective for characterizing the features of co-expressed gene modules [36], each of which is represented by a color classifier. Here, the correlation values were raised by a power of 12 to satisfy scale-free criteria [27]. The minimum module size was set to 40 genes and the height for merging modules was set to 0.25, which required at least 25% dissimilarity among modules in expression. We identified a total of 18 modules (Additional file 1: Table S1), each summarized by its eigengene (ME, defined as the first principal component of the standardized expression values [37]). The significance of module eigengene-phenotype association (cell type, mutation status, and resting vs. depolarization) was evaluated by a linear regression model using the R lm function. Associations with FDR (Benjamini-Hochberg (BH) correction [38]) less than 0.05 was considered as significant. Genes were prioritized based on their correlation with the module eigengene (kME) [37]. The top connected genes (either kME >0.6 or the top 200, depending on which was smaller to facilitate visualization) were used to generate the module network plots via the R igraph package [39].

### Module preservation analysis

Module preservation analysis was performed to investigate if density and connectivity based network measures were preserved across data sets and conditions [40]. A Zsummary statistic was computed to aggregate various preservation measures, and a threshold of 2 based on 200 permutations was used to determine significantly preserved modules.

We first assessed the preservation of modules identified in combined case and control samples in two independent data sets: (1) expression profiles of differentiating primary human neural progenitor cells *in vitro* over 12 weeks (phNPCs) (GSE57595) [41], and (2) expression data from developing human cortex (post conception week 4 through 6 months after birth) from Kang *et al*. (GSE25219) [41,42].

### Differential expression

Differentiation-induced expression changes were assessed for cases and controls separately using the linear models in the R limma package [43]. The neural progenitors and neurons were paired if they were differentiated from the same iPSC clone and plated for differentiation in one experiment. The interaction effect was further evaluated using factorial designs implemented in limma. To be identified as showing dynamic expression changes upon differentiation in TS versus controls two criteria needed to be satisfied: (1) significant differential expression upon differentiation in either controls or TS, but not both; (2) a significant interaction effect between cell type (neural progenitor and neurons) and TS mutation status. The significance threshold was set at *P* <0.05 unless otherwise specified.

### Functional enrichment analysis

Functional enrichment analysis was assessed using GO-Elite Pathway Analysis [44]. Two enrichment analyses were performed on the genes of interest by assessing: (1) enriched Gene Ontology (GO) categories, and (2) enriched KEGG pathways. GO-Elite performs permutations to obtain over-representation Z scores and enrichment *P* values for each GO term. In our analysis, we performed 10,000 permutations to evaluate enrichment significance. The background was set to the total list of genes expressed in this data set. GO categories with a permuted *P* <0.05 were reported.

### Gene set over-representation analysis

A one-sided Fisher exact test was performed to assess over-representation of module genes in other gene sets using the R function fisher.test. Depolarization-associated gene lists were curated from two publications (McKee *et al*. [45] and Kim *et al*. [46]). The ASD susceptibility genes were curated from the SFARI gene database [47]. Genes categorized as Syndromic (S) and those with associated scores in the range of 1 to 4 were used in our analysis. The ASD-associated co-expression modules asdM12 and asdM16 were obtained from Voineagu *et al*. [33]. The ID-associated genes were curated from four reviews [48-51] resulting in 401 genes as reported in Parikshak *et al*. [34].

### Transcription factor binding site (TFBS) enrichment analysis

TFBS enrichment analysis was conducted by scanning the promoter sequence of the genes in the analyzed modules for enrichment of known transcription factor binding motifs using the Clover algorithm [52]. For every gene, we considered 1,000 bp upstream of its transcription start site as the candidate promoter region. The putative binding motifs were obtained from TRANSFAC [53,54] in the format of position weight matrix. To comprehensively evaluate the statistical significance of the enrichment results, we utilized three different background datasets: 1,000 bp sequences upstream of all human genes, human CpG islands and the sequences of human chromosome 20. We calculated the enrichment *P* values from the null distribution generated by repeatedly drawing 1,000 random sequences of the same length from the background sequences. Significant events were defined at *P* <0.05 across all three backgrounds.

To confirm the validity of the predicted motif enrichment, we determined if existing chromatin immunoprecipitation (ChIP) data for transcription factors supported the predicted binding sites. The ChIP data sets were obtained from ENCODE [55,56] and ChIP Enrichment Analysis resource (ChEA) [57]. We reported the number of predicted binding targets that could be verified by corresponding transcription factor ChIP data from any tissues or cell lines where available. Statistical significance was evaluated by assessing the cumulative hypergeometric probability using phyper function in R. The population size was defined as the total number of genes expressed in this data set.

## Results

### Network construction and module detection

**Figure 1:**
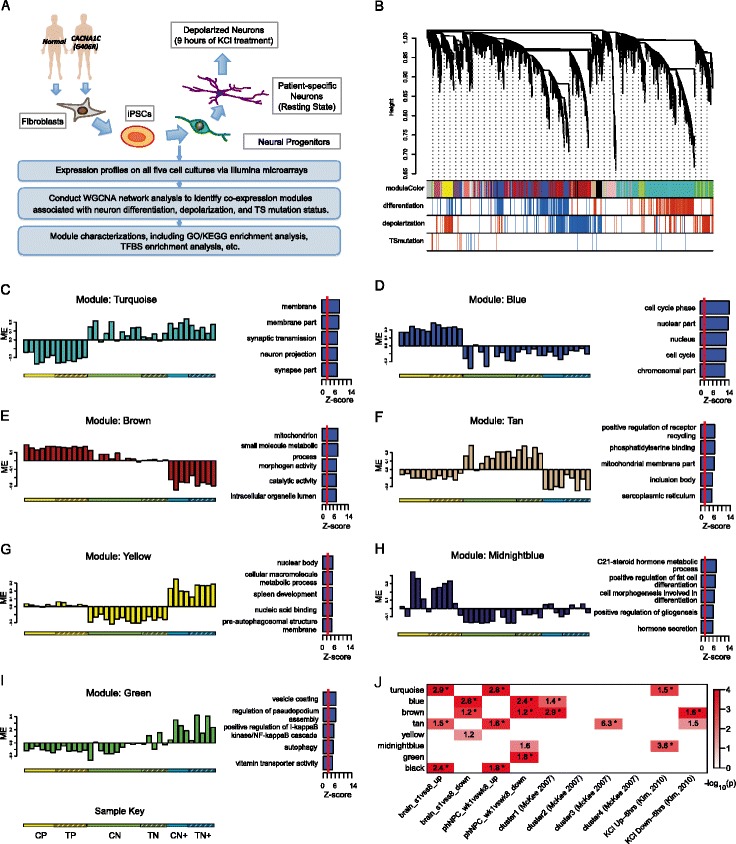
**WGCNA identifies co-expression modules associated with neuronal differentiation and depolarization. (A)** Flowchart illustrating the experimental procedures and expression analysis. **(B)** Hierarchical clustering of genes based on gene co-expression pattern across progenitors, neurons at rest, and after depolarization. Identified co-expression modules were represented by color classifiers, noted across the top of the dendrogram. The ‘differentiation’, ‘depolarization’, and the ‘TSmutation’ color bars represent the correlation values between gene expression and three biological traits: differentiation, depolarization, and *CACNA1C* G406R mutation status, respectively. Red signifies upregulation, while blue signifies downregulation. Only the genes with a trait correlation larger than 0.5 or smaller than -0.5 are marked in the plot. **(C-I)** Module eigengene patterns and enrichment scores of the top five enriched GO categories for module **(C)** turquoise, **(D)** blue, **(E)** brown, **(F)** tan, **(G)** yellow, **(H)** midnightblue, and **(I)** green. Samples are ordered by control progenitors (CP), patient progenitors (TP), control resting neurons (CN), patient resting neurons (TP), control depolarized neurons (CN+), and patient depolarized neurons, as illustrated by the key at the bottom. **(J)** Module-level enrichment for previously identified differentiation- and depolarization-associated gene sets curated from: (1) Kang *et al*. developing postmortem human brain [41,42]; (2) Stein *et al*. phNPC expression study (phNPC_wk1vswk8_up/down) [41]; (3) McKee *et al*. [45]; and (4) Kim *et al*. [46] KCl-induced depolarization expression studies. ‘brain_s1vss8_up/down’ represent the genes that are either up- or downregulated between stage 1 vs. stage 8, while ‘phNPC_wk1vswk8_up/down’ are the group of the genes up- or downregulated between week 1 vs. week 8 differentiation, as defined in the paper [41,42]. Cells are colored to reflect enrichment significance with ceiling of 10^-4^. Enrichment odds ratios are shown in the table if the *P* <0.05 (*FDR <0.05).

To elucidate the transcriptional changes relevant to TS mutation at key stages, we constructed a co-expression network based on the expression profiles of cortical neural progenitor cells (*N* = 12) and differentiated cortical neurons, both at rest (*N* = 15) and after KCl-induced depolarization (*N* = 9) (Figure 1A). As previously shown, iPSC lines were validated and the stages of neural differentiation *in vitro* were carefully characterized using a variety of immunocytochemical, physiological, and molecular assays, including Fluidigm Dynamic Arrays, patch-clamp recording, and live calcium imaging, to demonstrate the derived neurons expressed the appropriate molecular markers, were electrically active and fired action potentials [25] (Methods). Using a signed network analysis [27], we identified a total of 18 gene co-expression modules which were comprised of genes sharing highly similar expression patterns across samples. As shown in Figure 1B, genes that clustered into modules based upon co-expression also shared functional annotations, indicating that they participate in common biological processes.

We next assessed the reproducibility and generalizability of the network structure. We used module preservation analysis [40] to compare the identified modules with independent expression profiles from *in vivo* human brain development and *in vitro* neuronal differentiation of primary human neural progenitor cells (phNPCs) (Methods). Remarkably, the co-expression structure of 10 modules can be reproducibly identified in either of two independent expression datasets, differentiating phNPCs *in vitro* [41] or *in vivo* cortical development from post-conception week (PCW) 4 to 6 months after birth (Table 1; Additional file 2: Figure S1) [41,42]. Given the biological (different cells and tissues) and methodological differences (different RNA preparation and microarrays) between these studies, the correspondence with previous *in vivo* and *in vitro* expression data provides important validation of the transcriptional networks we identified in iPSC-derived neural cells.

**Table 1:** **Summary of co-expression modules associated with neuronal differentiation and depolarization, and TS mutation**

**Module color**	**Trait association (FDR <0.05)**			**Preservation**		**Top enriched GOs**	**Top 5 connected genes by kME**
	**Differentiation**	**Depolarization**	**TS mutation**	***In vivo***	***In vitro***		
Green yellow	↑	↑				Mammary gland epithelium development, midbrain - hindbrain boundary development, hemopoietic stem cell proliferation	*LMX1A*, *RSPO3*, *WLS*, *TM4SF1*, *ATF3*
Turquoise	↑	↑		Preserved	Preserved	Synapse part, synaptic transmission, neuron projection	*SNAP25*, *C12orf68*, *MYT1*, *MAP6*, *EEF1A2*
Black	↑	↑	↑			Nervous system development, nucleobase catabolic process, hydrolase activity	*RPS6KA2*, *ATP9A*, *KIAA1549L*, *FAM229B*, *AKT1*
Tan	↑	↓			Preserved	Mitochondrial membrane part, ribonucleoside triphosphate metabolic process, unfolded protein binding	*KIAA0368*, *ZNF706*, *SRI*, *C9orf169*, *OPA1*
Cyan	↑			Preserved		Skeletal muscle thin filament assembly, platelet-derived growth factor binding, actin-mediated cell contraction	*CAV1*, *SPP1*, *ME1*, *SPP1*, *VSX1*
Grey60	↑					*De novo*’ post-translational protein folding, mitochondrion, mitochondrial part	*TBCE*, *EBNA1BP2*, *CCT4*, *ASPH*, *AK2*
Midnight blue	↓	↑				Cell morphogenesis involved in differentiation, C21-steroid hormone metabolic process, positive regulation of fat cell differentiation	*TPD52L1*, *GJA1*, *BMP2*, *IL17RD*, *KRT15*
Yellow	↓	↑		Preserved		Nuclear body, positive regulation of cell growth, Golgi vesicle transport	*MRPS6*, *PCID2*, *PPP6C*, *GBA2*, *SETD4*
Brown	↓	↓		Preserved	Preserved	Intracellular organelle lumen, oxidation-reduction process, NADH dehydrogenase complex	*DOCK1*, *COMMD4*, *STRADB*, *SLC16A9*, *ORC3*
Blue	↓			Preserved	Preserved	Cell cycle phase, cell division, nuclear division	*NIF3L1*, *LIN28A*, *TEX10*, *AMMECR1*, *NCAPG*
Green		↑			Preserved	Vesicle coating, positive regulation of I-kappaB kinase/NF-kappaB cascade, autophagy	*NUP98*, *DNAJA1*, *DHX37*, *ETNK1*, *CGGBP1*
Red		↓	↑	Preserved	Preserved	Nucleobase metabolic process, nuclease activity, proteasome core complex	*USP13*, *PSMG1*, *RTN4IP1*, *PTCD2*, *PSMG1*
Light green			↑			tRNA processing, positive regulation of lipid metabolic process, response to virus	*CRYBB2*, *SNHG5*, *EXOC1*, *IFITM2*, *VAV3*
Magenta			↑			Type 1/2 fibroblast growth factor receptor binding; neuron recognition; growth factor activity	*CTSF*, *ZNF626*, *ZNF521*, *PLEKHA5*, *COL4A6*
Purple			↑	Preserved	Preserved	ncRNA processing; nuclear body, ribonucleoprotein complex biogenesis	*RRS1*, *RRP15*, *PUS1*, *NOLC1*, *ABCE1*
Light cyan			↓			Lytic vacuole, integral to organelle membrane	*TRAPPC2*, *ZNF177*, *HLA*-*A*, *ZNF559*, *RPS26P47*
Salmon			↓			Actin filament bundle assembly, regulation of establishment of protein localization in plasma membrane, regulation of type I interferon-mediated signaling pathway	*NDUFB11*, *MSN*, *UPRT*, *GPKOW*, *TSR2*

The top five connected genes ranked by kME and the top three enriched GO terms are listed for each module. Modules are labeled if they were preserved in independent *in vivo* and *in vitro* expression data sets [41,42] according to module preservation analysis [40] (Methods).

### Network analysis identifies differentiation and activity-dependent expression changes

We first sought to investigate if the identified co-expression networks recapitulate molecular processes related to neuronal differentiation and neuronal depolarization in general. We used the module eigengene (first principal component of the expression pattern of the corresponding module [27,37]) to summarize gene expression trajectories across samples, and evaluated the relationship of the 18 module eigengenes with differentiation and depolarization status. We found 10 modules strongly correlated with neuronal differentiation and nine modules significantly associated with KCl-induced neuronal depolarization (FDR <0.05), observed in both case and control cell lines (Table 1; Additional file 2: Figure S2). Representative examples with module eigengene trajectories and enriched GO terms are shown in Figure 1.

Importantly, as highlighted above, we found that many of differentiation and depolarization associated modules were present in independent *in vivo* and *in vitro* expression data sets (Table 1), providing independent validation for these *in vitro* iPSC-derived networks. For instance, as compared to *in vivo* human fetal brain transcriptional networks, the modules corresponding to iPSC-derived cortical neurons faithfully recapitulated biological processes driving *in vivo* cortical development, including neurogenesis and differentiation (blue and yellow modules), axonogenesis and dendrite growth (turquoise), as well as synaptogenesis (turquoise and green yellow modules) (Figure 1; Table 1; Additional file 2: Figure S3) [41,42]. Additionally, the genes within depolarization-associated modules (brown, tan, green yellow, and midnight blue modules) demonstrated significant overlap with previously defined depolarization-associated gene sets defined in mouse cortical neurons [46] and human neuroblastoma cells (IMR-32) [45] before and after KCl treatment (Figure 1J). In particular, the two modules downregulated upon depolarization (brown and tan) were enriched for GO categories related to mitochondria, suggesting altered energy consumption upon prolonged neuron depolarization, in agreement with McKee *et al*. in human neuroblastoma IMR-32 cells [45]. Together these findings demonstrate the power of WGCNA in identifying generalizable, functionally important gene modules.

Interestingly, a subset of modules was enriched for genes affected by both differentiation and depolarization (Figure 1J; Additional file 3: Table S2), which could provide a molecular basis for modulation of neuronal differentiation by depolarization [58-60]. For instance, the brown module, whose module eigengene showed dramatic down-regulation in depolarized neurons, also followed a significant decrease with neuronal differentiation (Figure 1E). On the other hand, the module eigengenes of the yellow, tan, black, and midnight blue modules, showed opposite directions upon differentiation as compared with depolarization (Figure 1F-H). These observations were consistent with the notion that neuronal plasticity can recapitulate processes involved in neuronal development [61-63].

### Gene co-expression modules dissect pathways related to different aspects of TS symptoms

**Figure 2:**
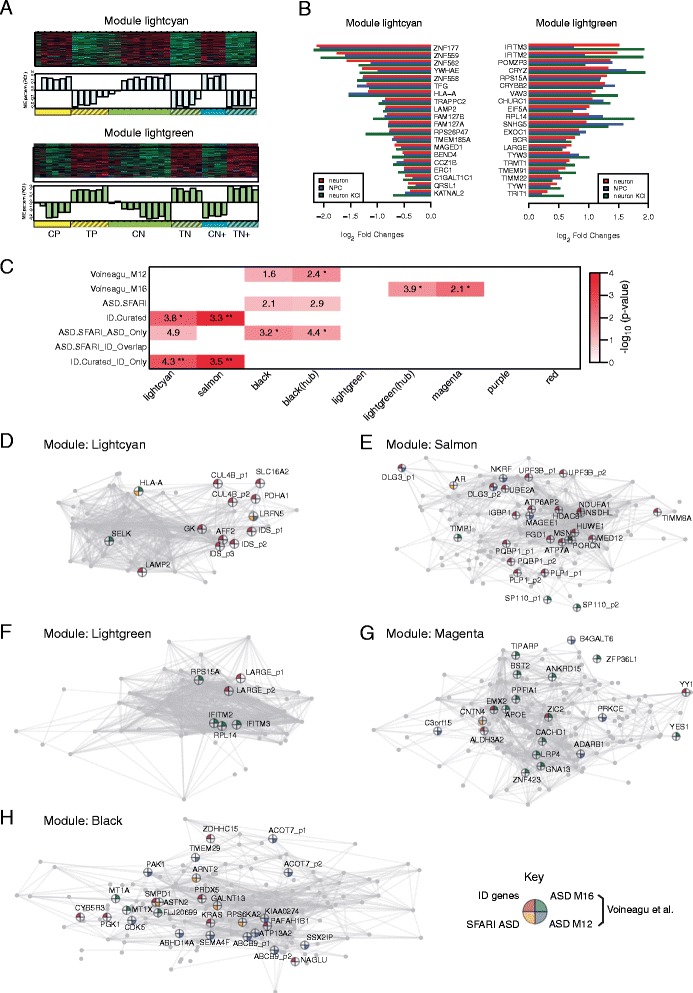
**Gene co-expression modules associated with the TS mutation. (A)** Heatmap showing expression pattern of the module genes and a barplot showing the module eigengene pattern across samples for the light cyan and light green modules. **(B)** Log_2_ transformed fold changes of the top 20 connected genes in the light cyan and light green modules in cases as compared to controls at the three experimental stages. **(C)** Module-level enrichment for previously identified ASD and ID associated genes. Enrichment odds ratios are shown in the table if the *P* <0.1 (*P <0.05, **FDR <0.05). **(D-H)** Visualization of the co-expression network among the top connected genes (kME >0.6 or top 200 depending on which one is smaller) based on multidimensional scaling of their pairwise co-expression correlations in the **(D)** light cyan, **(E)** salmon, **(F)** light green, **(G)** magenta, and **(H)** black module. Genes with multiple probes are labeled separately. Pie chart: ID susceptibility genes (red); ASD susceptibility genes from the SFARI database (yellow) [47]; genes in the Voineagu *et al*. asdM12 module (purple) [33]; genes in the Voineagu *et al*. asdM16 module (green) [33]. Only the top 1,000 connections are shown in each module.

Next, we asked if we could identify modules associated with TS mutation status, which would provide insight into dysregulation of molecular networks in TS and disease pathophysiology. By comparing the module eigengene patterns across patient and control cells, we identified seven modules that were significantly associated with the TS mutation (FDR <0.05). Remarkably, the top two most disease correlated modules (light green and light cyan; R-square >0.8; Figure 2A and B) included dysregulated genes previously implicated in neurodevelopmental diseases, such as *YWHAE* (Miller-Dieker Syndrome) [64], *ERC1* (12p13.31 deletion associated developmental delay) [65], and *VAV3* (schizophrenia) [66] (Figure 2B).

A further critical question is: to what extent these TS-associated modules can inform us about the molecular mechanism of TS-related abnormalities? Here, we investigated every module by GO/KEGG enrichment analysis and performed over-representation analysis with respect to curated disease associated genes (Methods). We observed striking enrichment of known ID susceptibility genes [48-51] in two downregulated modules, light cyan and salmon (Figure 2C; Additional file 3: Table S3). Specifically, in the light cyan module, seven ID genes were identified: *LAMP2*, *GK*, *IDS*, *CUL4B*, *AFF2*, *PDHA1*, and *SLC16A2* (Fisher’s exact test: enrichment odds ratio (OR) = 3.8; *P* = 0.004). More importantly, these seven ID candidate genes form a sub-cluster within the light cyan module (Figure 2D), suggesting their tight functional dependence. Moreover, this module was enriched for GO categories involved in organelle membrane, and the KEGG pathway of ubiquitin mediated proteolysis, which agrees with previous reports of the causal relationship between impaired proteasomal activity and cognitive disorders, including ID [67]. The salmon module, which contained genes downregulated in cells carrying the TS mutation, was even more enriched for ID susceptibility genes, containing 18 genes known to cause ID (OR = 3.3, *P* = 3e-05) (Figure 2E). Together the identification of these two downregulated modules provides an unbiased starting point based on gene expression for exploring the molecular connections between the TS mutation and the molecular mechanisms of ID [9].

In contrast to the salmon and light cyan modules enriched for ID genes, the black downregulated module was enriched for ASD candidate risk genes curated from the SFARI gene database [47] (Figure 2C; Additional file 3: Table S4). Six known ASD candidate susceptibility genes were identified in the black module (OR = 2.5, *P* = 0.04), and three of them, *ASTN2*, *ARNT2*, and *RPS6KA2*, were hubs (Figure 2H). More importantly, the top connected genes in the black module (kME >0.7) significantly overlapped with a previously defined co-expression module, called asdM12, identified via unbiased transcriptome analysis in postmortem ASD brains (OR = 2.4, *P* = 0.02) [33], but not preserved in control tissues. asdM12, which contains genes involved in synaptic development and function, was downregulated in cerebral cortex from ASD subjects, in parallel with the observed decrease of the black module genes in TS observed here. Consistent with asdM12 annotation, the black module also was enriched for postsynaptic density (PSD) associated genes [68] (OR = 1.9, *P* = 0.001) that are critical regulators of synaptic signaling and plasticity. These observations suggest convergent synaptic dysfunction in this monogenic form of ASD caused by TS studied here and idiopathic ASD more broadly. The non-overlapping relationship of known ID and ASD susceptibility genes to specific modules was also consistent with recent work demonstrating differing *in vivo* expression patterns of genes causing these two clinically distinct conditions [34].

We next evaluated the upregulated modules in TS neurons. As shown in Figure 2C, the light green and magenta modules show significant overlap with asdM16, a module of genes upregulated in ASD postmortem brain [33] (Additional file 3: Table S5). The biological functions enriched in this module include immune response, which is consistent with immune dysfunction observed in TS [9,10,69]. In particular, 18 genes in the ASD postmortem asdM16 module were identified in the magenta module, and four were identified in the light green module (Figure 2F-G). Remarkably, *IFITM2* and *IFITM3*, two interferon response genes that have been shown to be the hub genes in asdM16 [33], were also identified as hubs in this light green module, showing parallel dysregulation in ASD and TS patients. In the light green module, we also identified *INPP5E*, a gene involved in phosphatidylinositol signaling system and known to mobilize intracellular calcium. Mutation of this gene leads to Joubert syndrome, which is a rare monogenic condition with high penetrance for ASD [70-73]. In summary, both down- and upregulated modules in TS show changes parallel with those observed in postmortem brain of idiopathic ASD, consistent with the existence of convergent molecular pathways in multiple forms of ASD [74].

### Network analysis reveals differentiation defects in TS

**Figure 3:**
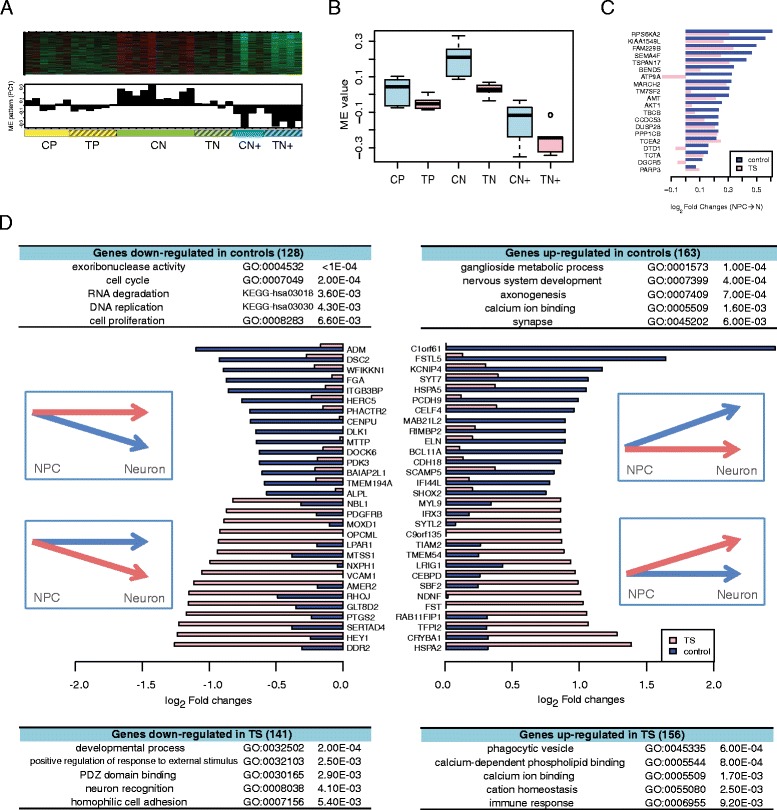
**Differentiation defects in TS cortical neural progenitors. (A)** Heatmap showing gene expression patterns in the black module and a barplot showing the corresponding module eigengene trajectory. **(B)** Boxplot comparing the module eigengene patterns between case cells vs. control cells at three experimental stages. **(C)** Barplot showing the log_2_ fold changes of the top 20 connected genes in the black module upon differentiation. Blue bars show the log_2_ fold changes in control samples, and pink bars represent the log_2_ fold changes in patient samples. **(D)** Differentiation-dependent gene expression changes in the patient progenitors. The differentiation-associated genes are categorized into four groups: (1) genes downregulated upon differentiation in controls but not in patients (top left); (2) genes that are upregulated upon differentiation in controls but not in patients (top right); (3) genes downregulated upon differentiation in patient cells but not in controls (bottom left); and (4) genes upregulated upon differentiation in patient cells but not in controls (bottom right). The log_2_ fold changes of the top 15 genes that show largest difference between cases and controls are shown in each group. The top enriched GO categories and KEGG pathways identified in each of the four gene groups are shown in the corresponding tables with enrichment *P* value attached.

We previously showed that the cell lines derived from TS patients had abnormalities in differentiation at the cellular level [25]. Here, we sought to investigate if we can use the unbiased transcriptomic approach to find the molecular mechanisms driving this differentiation deficit. By comparing the module eigengene expression patterns during neuronal differentiation across TS and control cells, we observed that the black module was upregulated upon differentiation in controls, but not in patient cells (Figure 3A and B). Comparison of expression fold changes of the top 15 connected genes in the black module during the progenitor to neuron transition are shown in Figure 3C, demonstrating the dramatic attenuation in differentiation related expression changes in patient versus control neurons. This parallels with the overlap of black module genes with asdM12, which as described above, is down regulated in post mortem ASD brain versus control [25].

To further characterize the differentiation abnormalities in the TS patient cells, we used a factor design to identify genes showing altered expression trajectories during differentiation in TS and control cells (Methods). We identified 1,155 genes with a significant interaction effect between developmental stage and disease status (*P* <0.05). By carefully evaluating their expression patterns in patient and controls samples (Methods), we further classified these genes into four categories: (1) genes downregulated upon differentiation in control but not in TS cells (128 genes); (2) genes upregulated upon differentiation in control but not in TS cells (163 genes); (3) genes downregulated upon differentiation in TS cells but not in control (141 genes); and (4) genes upregulated upon differentiation in TS cells but not in control (156 genes). The 15 genes with largest difference between cases and controls in each group are displayed in Figure 3D. As expected, the black module significantly overlapped with control-specific upregulated genes (hypergeometric test; *P* = 2.39e-6), again consistent with a defect in synaptic differentiation in TS.

We also found, not unexpectedly, that these four groups of genes with distinct disease and differentiation trajectories manifest distinct functional ontologies. For instance, genes that were downregulated in control neurons, but not in TS neurons, were enriched for functional categories related to cell cycle control, DNA replication, and cell proliferation (Figure 3D). This suggests a defect in the cell cycle of TS neural progenitors that could contribute to the corticogenesis defects we have previously demonstrated [25]. On the other hand, genes, such as *CTNNA2*, *SNCA*, and *SYT7*, exhibit control-specific upregulation pointing to pathways related to synaptic function, axonogenesis, and nervous system development (Figure 3D). Similarly, the genes exclusively down-regulated upon differentiation in TS patient cells were enriched for the GO categories of neuron recognition, PDZ domain binding, and homophilic cell adhesion, all involved in synaptic development, including *CADM1*, *FEZF2*, and *OPCML* (Figure 3D). In addition, the GO terms enriched among TS-specific upregulated genes were related to cation homeostasis control, and calcium ion binding activities, such as *AGTR1*, *ANXA7*, and *ITSN1* (Figure 3D), which were consistent with the biophysical dysfunction of the ion channels carrying the TS mutation. Taken together, our findings suggest a global effect of the *CACNA1C* G406R mutation on neuronal differentiation and point to specific pathways and genes that warrant further experimental study.

### TS associated co-expressed genes are co-regulated by calcium-dependent transcription factors

As shown in Paşca *et al*., increased [Ca^2+^]_i_ elevations were observed in TS-derived neural progenitors and neurons after depolarization [25]. Thus we investigated how the identified TS-associated expression features could be related back to the causal TS calcium channel mutation and corresponding changes in [Ca^2+^]_i_ signaling. It is known that calcium influx regulates activity-dependent gene expression through a hierarchical transcription network acting through multiple signaling cascades [3,75]. While simple lists of up- and downregulated genes may not provide power to identify regulatory mechanisms, we hypothesized that these tight co-expression modules would reflect calcium-dependent co-regulation. To test this, we performed transcription factor binding site (TFBS) motif enrichment analysis on the seven TS associated modules to investigate whether those modules were enriched for any calcium-dependent transcriptional regulators (Methods).

**Figure 4:**
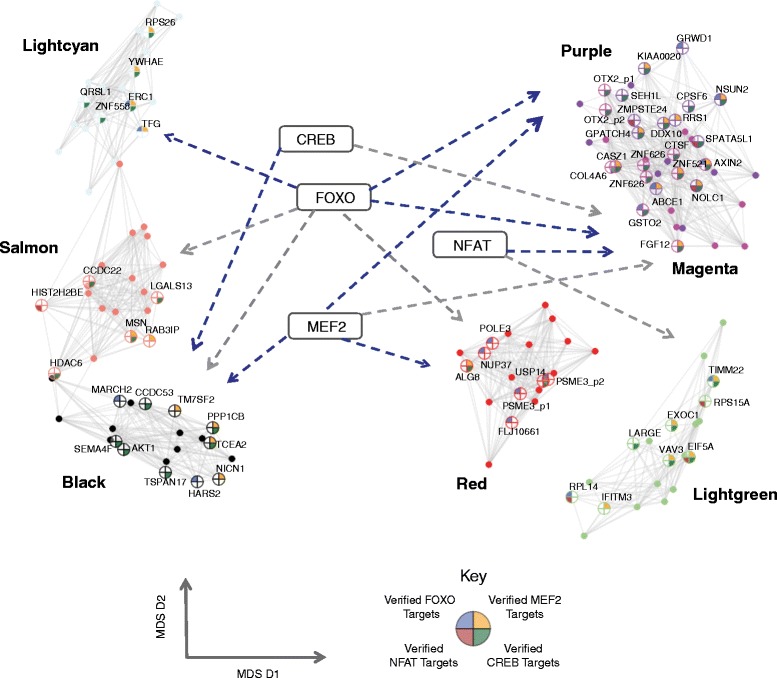
**Timothy syndrome-associated modules are regulated by known calcium-dependent transcription factors.** The TS-associated modules are enriched for targets regulated by known calcium-dependent transcription factors, MEF2, NFAT, CREB, and FOXO, which are listed in the middle of the figure. Dashed lines indicate significant enrichment of the designated transcription factor binding targets (*P* <0.05) in the corresponding modules (blue lines indicate *P* <0.01). The top 20 connected genes in each module are plotted according to the multidimensional scaling of their pairwise co-expression correlations, so that co-expressed genes are clustered to each other. Genes are connected if their pairwise correlations are higher than 0.7. Pie chart: ChIP-seq verified FOXO targets (blue); ChIP-seq verified NFAT targets (red); ChIP-seq verified CREB targets (green); ChIP-seq verified MEF2 targets (yellow). Genes with multiple probes are labeled separately.

For each module, we identified a set of transcription factor binding sites enriched within a 1 kb window upstream of the transcription start site, providing strong evidence for the co-regulation hypothesis. Moreover, this TFBS analysis identified four important calcium-regulated transcription factor families in the TS related modules: NFAT [19,20], MEF2 [17,18], CREB [21-24], and FOXO [29-32]. Among them, FOXO proteins, which regulate neuronal polarization and positioning [76] and synaptic function and memory consolidation [30], have binding targets enriched in six out of seven TS-associated modules (light cyan, salmon, magenta, black, purple, and red) (Figure 4). A total of 1,249 predicted targets were identified in these six modules, and 229 of them were validated through data from chromatin immunoprecipitation (ChIP) experiments [55-57], providing a significant validation of the bio-informatic predictions (hypergeometric test; *P* = 7.73E-12; Methods).

The other TF enrichments were limited to smaller subsets of modules. Perhaps most remarkable is the enrichment of NFAT targets in two modules upregulated in TS, magenta and light green (also asdM16 associated, which is upregulated in idiopathic ASD [33]; Figure 4). Four of the five members in this protein family, NFATc1, NFATc2, NFATc3, and NFATc4, are known to be regulated via calcium signaling [19,20,77,78]. Forty-two genes (68%) in the light green module and 262 genes (82%) in the magenta module were predicted to contain at least one NFAT binding site in their promoter regions. No ChIP data are available from developing neurons, but in lymphoblasts [55,56], we were able to observe direct binding to 25 neuronal targets (hypergeometric test; *P* = 2.02E-2). Taken together, these results suggest that the two upregulated modules (light green and magenta), which also represent the convergence of TS and ASD at the level of gene expression, are likely to be mediated via the calcium/NFAT signaling pathway.

MEF2, a well-studied calcium or neuronal activity dependent transcription factor family number [3,17,18], was enriched in the promoter regions within genes contained in four TS associated modules: black (downregulated in TS and with activity, asdM12 associated), magenta (upregulated in TS, asdM16 associated), purple, and red (Figure 4). Of the four different MEF2 family members, MEF2A, MEF2B, MEF2C, and MEF2D, three (MEF2A/C/D) had their binding sites enriched in all four modules. Specifically, 309 genes (76.5%) in the black module, 246 genes (77%) in the magenta module, 214 genes (75%) in the purple module, and 354 genes (77%) in the red module contain at least one MEF2 binding site. Using experimental ChIP-seq and ChIP-chip data [55-57], we were able to validate a total of 358 predicted MEF2 binding targets (hypergeometric test; *P* = 6.58E-20) (Methods). Remarkably, MEF2A and MEF2C were previously reported to have binding sites enriched in idiopathic ASD associated co-expression modules, consistent with the notion of involvement of activity-dependent dysregulation in idiopathic ASD pathogenesis [3,34].

Targets of two CREB proteins, CREB1 and CREB2, were also enriched in the black (downregulated in TS and with activity, asdM12 associated), and magenta (upregulated in TS, asdM16 associated) modules (Figure 4). CREB transcription factors bind to the cAMP-responsive element (CRE), and are regulated by calcium influx [1,21-23,79]. One hundred and sixty-one (40%) genes in the black module and 134 genes (42%) in the magenta module were predicted to have at least one CREB binding site, 164 of which could be validated through available ChIP experiments [55-57] (hypergeometric test; *P* = 4.15E-63) (Methods). Moreover, we observed overlap of the predicted targets between the CREB-transcriptional machinery and the MEF2 proteins (Additional file 2: Figure S4), although their binding motifs are quite different. One hundred and forty-eight genes have at least one predicted binding site for both MEF2 and CREB proteins in the black module and 123 (39%) in the magenta module, respectively, strongly implicating a synergistic interaction between the two pathways upon calcium influx.

Taken together, our results not only demonstrate significant co-regulation among the co-expressed genes, but also provide specific regulatory links for associating distinct co-expression modules. More importantly, these findings provide a path for bridging the observed downstream transcriptional alterations back to the mutation in the L-type calcium channel Cav1.2 via their regulation by calcium dependent transcription factors.

## Discussion

TS is a rare and complex disorder characterized by a broad spectrum of phenotypic abnormalities. There are few TS patients available for study and the data used here represent the only gene expression data set available in this disorder. Here, we studied multiple cell lines from independent differentiation experiments with four control iPSC clones from two normal subjects, four TS iPSC clones from one TS patient carrying a dominantly acting mutation, and one human embryonic stem cell line (H9; additional control) to mitigate the concerns about the effect of induction of pluripotency, or other confounding factors that could bias the results. Through analysis of data from iPSC-derived cortical neural progenitors and neurons, we identified distinct gene expression modules that are associated with human neuronal differentiation and neuronal depolarization across all conditions. We further demonstrate that the networks identified in control and TS iPSC-derived neural progenitors and neurons can be validated in independent *in vitro* and *in vivo* data sets. Moreover, we identified several co-expression modules that were correlated with TS mutation status, highlighting potential molecular pathways that may contribute to distinct phenotypic aspects of TS. Remarkably, by integrating the transcriptional networks defined by our co-expression analysis with TFBS enrichment analysis, we showed that the TS-associated expression changes are co-regulated by a set of calcium-dependent transcriptional factors. Furthermore, many of the specific genes and processes identified here in this monogenic condition overlap with those identified in postmortem brains from patients with idiopathic ASD. Consequently, the module hub genes and the identified transcription factors provide an important source of new candidate genes for therapeutic targeting. These intriguing results indicate that study of additional TS patient lines, when available, will be valuable.

As with other single gene disorders, how a mutation in a single gene yields such pleotropic CNS phenotypes provides a significant challenge [80]. Here, our bio-informatic analysis links specific molecular pathways perturbed in TS neurons to different aspects of TS, including ID, alterations in immune response, and behavioral phenotypes overlapping with ASD [8-10]. The identification of modules highly enriched for genes that either cause or increase risk for ID and ASD, provides new avenues to investigate the pathways that may mediate divergence between these disorders [34]. In particular, the enrichment for genes that were dysregulated in idiopathic ASD brain (asdM12 and asdM16) [33] demonstrates the existence of previously suggested convergent molecular pathways in idiopathic ASD in this monogenic highly penetrant form of ASD [33-35]. In parallel with recent findings, our analysis also indicates distinct modules associated with ASD (black, light green, and magenta) and ID (light cyan, salmon), consistent with divergent molecular mechanisms for ASD and ID [34]. Our analysis also prioritizes important gene sets (module hub genes) and pathways for further analyses. These genes can be helpful to understand how diverse genetic syndromes converge on ASD and how they are modulated. For example, two interferon response genes, IFITM2 and IFITM3, that are dysregulated in ASD brains [33] and the light green module in TS, as well as RPS6KA2 and AKT1 in the black module, highlight potential convergent molecular links between TS and ASD that warrant future experimental investigation.

Network analysis allowed us to determine disease-associated alterations at the level of transcriptional co-regulation. TFBS enrichment analysis prioritized several candidate transcription factors as putative regulators of disease-associated modules, most of which could be confirmed by experimental data. These findings provide direct evidence for our hypothesis that module gene co-regulation reflects transcription factor binding. More importantly, our analysis identified four known calcium-dependent gene transcription factor families that regulate key genes within these modules: FOXO [29-31], NFAT [19,78], MEF2 [17,18], and CREB [21-23]. Moreover, by showing the overlap of the TF targets within modules, our analysis also implicates coordination among those calcium-dependent transcriptional regulation pathways. In particular, we predict a synergistic effect between MEF2 and CREB proteins in TS cells, consistent with the observation that phosphorylation of both MEF2 and CREB proteins leads to recruitment of CREB-binding protein (CBP) to activate downstream transcription [81]. Lastly, since several modules regulated by these calcium-dependent pathways are also associated with ASD, these data support previous suggestions that dysregulation of activity-dependent signaling plays a more general role in ASD pathogenesis [3].

Also, of note, our analysis also highlights the potential role for RSK (ribosomal S6 kinase) proteins as putative regulators of genes in the black module. RSK proteins have been implicated in disorders of cognition and behavior, and mutations in RSK2 lead to Coffin-Lowry syndrome, an X-linked dominant genetic disorder that causes severe mental problems [82]. RPS6KA2 (also known as ribosomal s6 kinase 3, RSK3), RPS6KA4, and AKT1, all kinases that are known to regulate CREB [79,83-86], were identified in the black module, where RPS6KA2 and AKT1 were hub genes. Additionally, several known RSK and AKT substrates were found in the black module, including GSK3A, BEX1, CTNND2, and PAK1, which were centrally located in the protein-protein interaction network of the black module (Additional file 2: Figure S5). These observations lead us to speculate that RSK3/AKT1/CREB have key regulatory roles in the black module, and that downregulation of the black module in TS samples is due to downregulation of RSK/AKT pathways, a hypothesis that can be directly tested through experimental investigation.

Neuronal development signaling and plasticity depends on electrical activity [3,61-63]. For instance, KCl-mediated depolarization of neurons changes the chromatin accessibility of several differentiation-associated genes, such as *NCAM* and *TH*, and can subsequently alter the differentiation path of neurons [58,60]. However, neuronal depolarization has rarely been investigated at the genome-wide scale in human derived neural progenitors and neurons. Here, we identified five modules (brown, tan, yellow, midnight blue, and black) that were highly correlated with both differentiation and depolarization, providing a molecular network connecting these processes. An illustrative example is the black module, which is associated with the TS mutation, and was upregulated upon differentiation and downregulated upon depolarization. Importantly, as implicated by the black module trajectory, TS derived neural progenitors exhibited significant differentiation deficits, strongly implicating the involvement of Ca_v_1.2 in neural development. This is supported by changes in several genes involved in cation homeostasis control, including *AGTF1*, *ANXA7*, *CD55*, *HMOX1*, *SFXN4*, *SLC11A2*, *SLC39A14*, and *SLC4A11*, which were exclusively upregulated in TS progenitors upon differentiation, consistent with large-scale changes in the molecular networks associated with differentiation in TS.

## Conclusions

Our results define a transcriptional network outlining a shared molecular basis for cortical neural differentiation and neuronal depolarization, but also implicate dysregulation of these common molecular pathways in TS pathogenesis. We show that several of these molecular pathways dysregulated by this specific Ca_v_1.2 mutation are shared with idiopathic ASD based on comparison with data from *in vivo* brain gene expression. By defining the core molecular changes downstream of the Ca_v_1.2 mutation and its transcriptional regulators, this work illustrates how an integrative approach can be applied to functionally characterize transcriptional co-regulation under physiological and disease states, and to generate hypotheses to drive further mechanistic experimental investigation.

## Additional files

Additional file 1: Table S1. Module assignment and module membership (kME) for all probes that passed the robust expression criteria. Additional file 2: Figure S1. Module-based preservation in independent expression data sets from human brain development and neuron differentiation *in vitro*. **Figure S2.** Module eigengene correlation with neuron differentiation and depolarization, as well as the TS mutation status. **Figure S3.** Module-level enrichment for *in vivo* defined modules during fetal brain development [41,42]. **Figure S4.** Overlap of MEF2 and CREB predicted targets. **Figure S5.** A protein-protein interaction network comprises the top connected genes in the black module (kME >0.7) [87]. Additional file 3: Table S2. Statistic significance of module-level enrichment for previously identified differentiation- and depolarization-associated gene sets. Odds ratio, *P* value, and Benjamini-Hochberg (BH) corrected FDR for significant overlap at *P* <0.05 are shown. **Table S3.** Statistic significance of module-level enrichment for previously identified ASD and ID associated genes. Odds ratio, *P* value, and BH corrected FDR for significant overlap at *P* <0.1 are shown. **Table S4.** A table listing ID susceptibility genes in the light cyan and salmon modules. **Table S5.** A table listing ASD associated genes in the black, light green, and magenta modules.

## Abbreviations

ASD: Autism spectrum disorders
ChIP: Chromatin immunoprecipitation
GO: Gene ontology
ID: Intellectual disability
iPSC: Induced pluripotent stem cell
ME: Module eigengene
PCW: Post-conception week
phNPC: Primary human neural progenitor cells
TFBS: Transcription factor binding site
TS: Timothy syndrome
WGCNA: Weighted gene co-expression network analysis
