# Supplementary material for: Alteration in basal and depolarization induced transcriptional network in iPSC derived neurons from Timothy syndrome
Source: Genome Med. 2014 Oct 10;6(10):75. doi: 10.1186/s13073-014-0075-5 (PMC4213483; doi:10.1186/s13073-014-0075-5)
Supplement: Additional file 2: Figure S1. — Module-based preservation in independent expression data sets from human brain development and neuron differentiation in vitro. Figure S2. Module eigengene correlation with neuron differentiation and depolarization, as well as the TS mutation status. Figure S3. Module-level enrichment for in vivo defined modules during fetal brain development [41,42]. Figure S4. Overlap of MEF2 and CREB predicted targets. Figure S5. A protein-protein interaction network comprises the top connected genes in the black module (kME >0.7) [87]. [file 13073_2014_75_MOESM2_ESM.pdf]

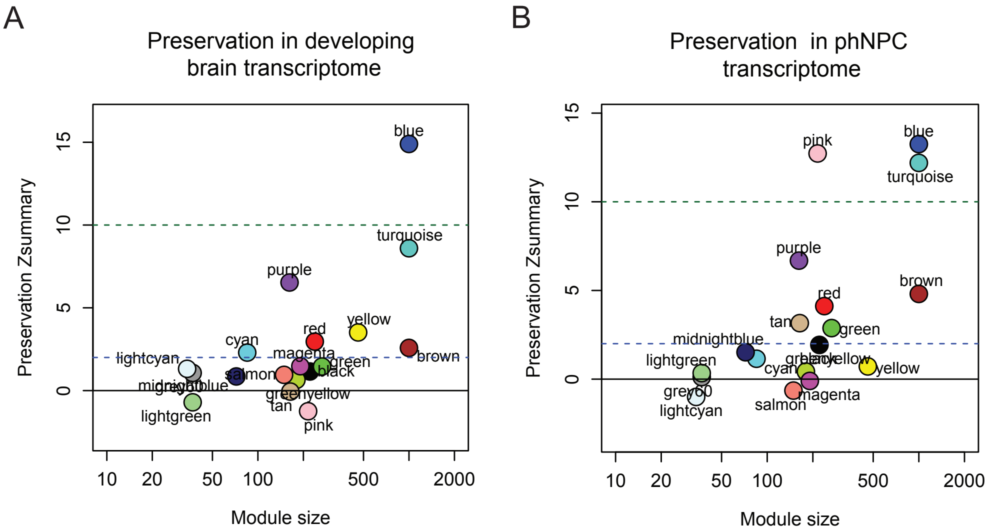

Figure S1. Module-based preservation in independent expression data sets from human brain development and neuron differentiation *in vitro*. A Zsummary statistic was computed to aggregate various preservation measures, and a threshold of 2 based on 200 permutations was used to determine significantly preserved modules. (A) Module preservation analysis of our identified modules show significant preservation of 7 modules in expression data profiling *in vivo* cortical development from 4 PCW to 6 month after birth [1, 2]. (B) Module preservation in an independent *in vitro* expression dataset, which profiled differentiating primary human neural progenitor cells (phNPCs) over 12 weeks and identified 8 significantly preserved modules [2].

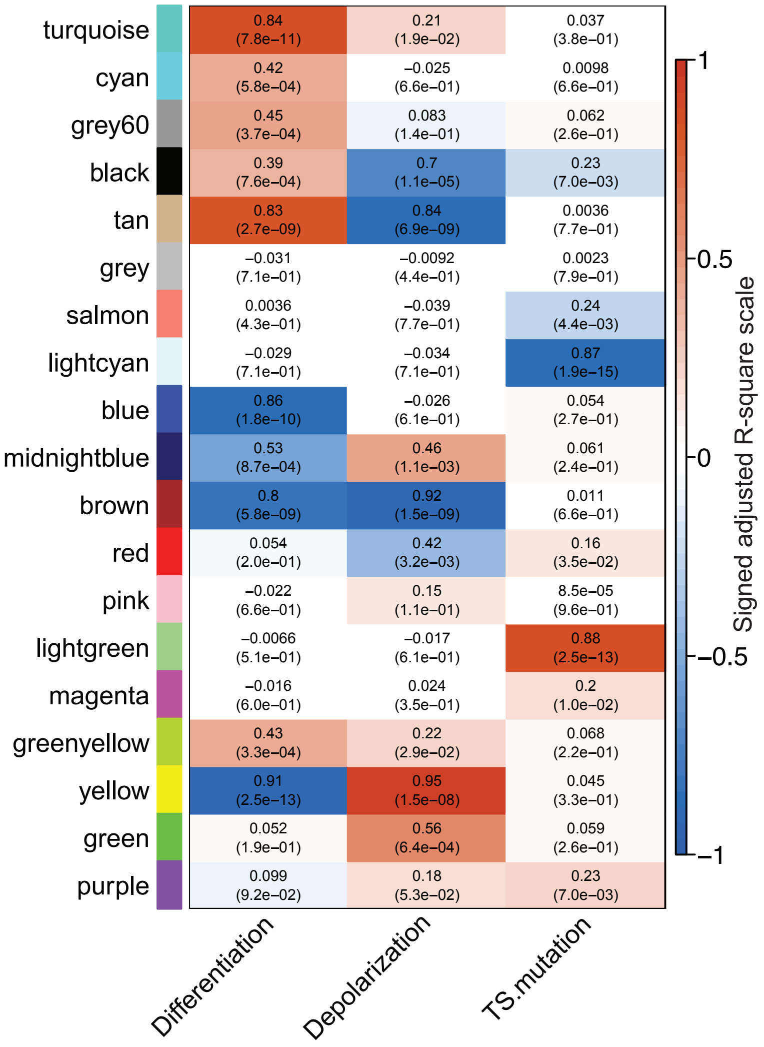

Figure S2. Module eigengene correlation with neuron differentiation and depolarization, as well as the TS mutation status. In each cell, the R-square value for association with module eigengene is shown on the top, and the association FDR (Benjamini–Hochberg (BH) correction [3]) is shown at the bottom. The cells are colored by signed adjusted R-square values with red and blue representing positive and negative correlation, respectively.

A

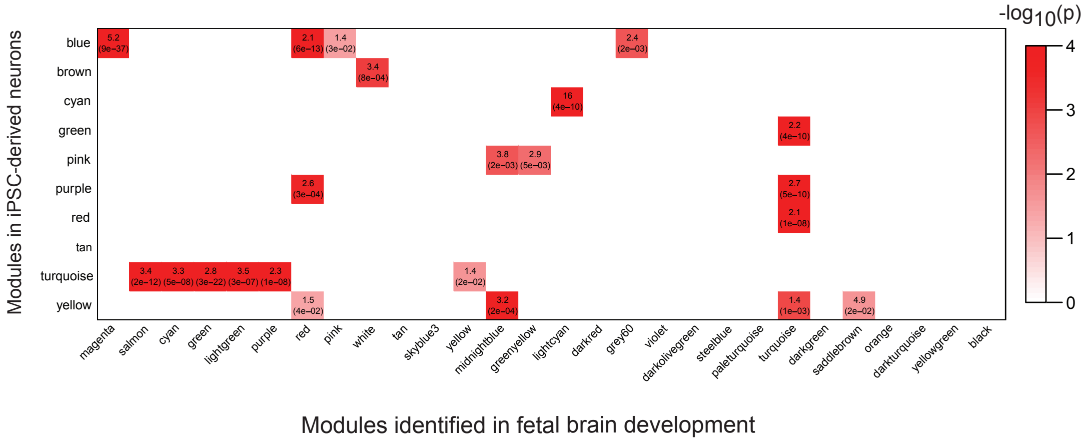

B

Brain Modules	Biological Processes Associated with Module as in Stein et al.
Magenta	Mitosis and cell cycle regulation of neural progenitors
Salmon	Glutamatergic synaptic transmission, axon and dendrite development
Cyan	Synapse assembly and vesicle transport by actin/microtubule motors
Green	Glutamatergic synaptic transmission, axon and dendrite development
Lightgreen	GABAergic synaptic transmission and synaptic vesicle exocytosis
Purple	Axon guidance and GTPase activity
Red	Mitosis, RNA processing and RNA splicing
Pink	Neural progenitor proliferation and gliogenesis
White	Undefined
Yellow	Synaptic transmission, gliogenesis and neuron microglia interaction
Midnightblue	Histone modification and chromatin remodeling
Greenyellow	RNA binding
Lightcyan	Extracellular matrix and basement membrane, blood vessel development
Grey60	RNA splicing, DNA repair and cell cyle
turquoise	Ubiquitin proteolysis, RNA processing and splicing, reg. gene expression
Saddlebrown	Undefined

Figure S3. Module-level enrichment for *in vivo* defined modules during fetal brain development [1, 2]. (A) Enrichment odds ratios (top) and BH corrected FDR [3] (bottom) are shown if the FDR p value is less than 0.05. Only the modules preserved in either differentiating phNPC *in vitro* or *in vivo* cortical brain were evaluated. (B) Functional annotation of the brain modules that were significantly overlapped with the modules defined in iPSC-derived neurons as in Stein *et al.* (2014) [2].

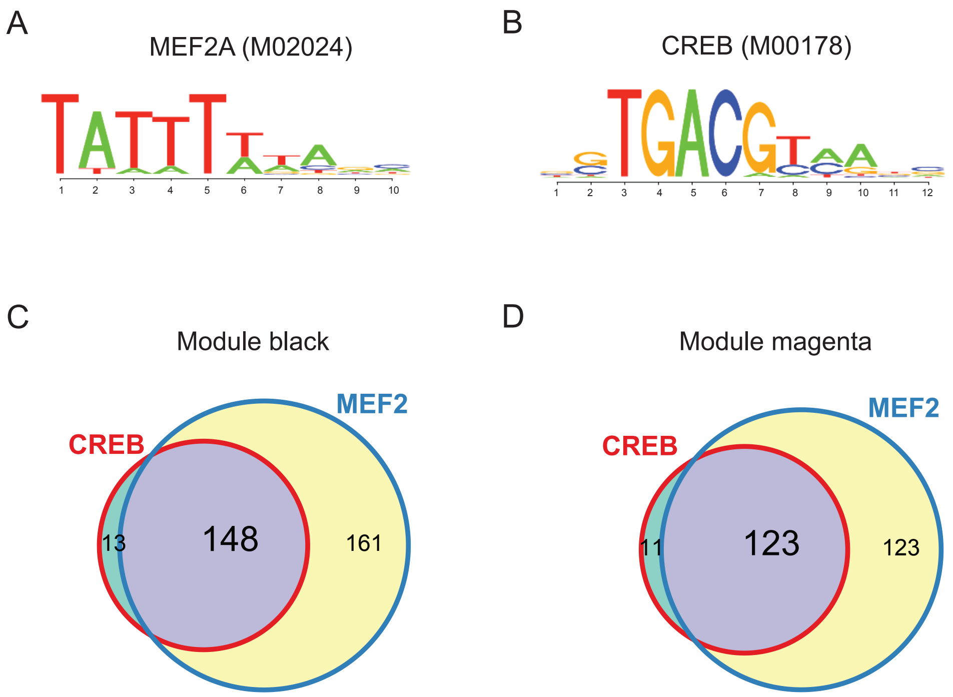

Figure S4. Overlap of MEF2 and CREB predicted targets. (A, B) Sequence logo plot for transcription factor (A) MEF2A and (B) CREB binding motifs curated from the TRANSFAC database [4, 5]. (C, D) Overlap of the predicted binding targets of CREB and MEF2 in the (C) black module and (D) magenta module.

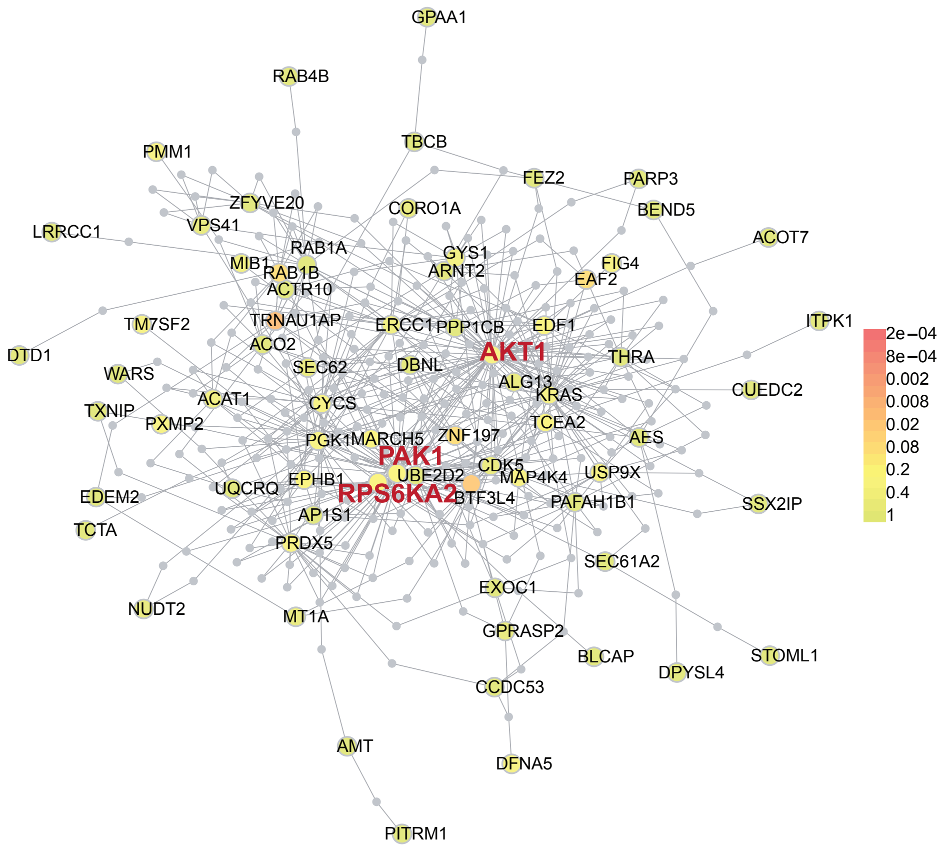

Figure S5. A protein-protein interaction network comprises the top connected genes in the black module (kME>0.7) [6]. Analysis was performed with Disease Association Protein-Protein Link Evaluator (DAPPLE), which complies the protein-protein interactions from "inWeb" database, and builds both direct and indirect networks among the proteins encoded by seed genes. The seed genes are colored by the probability that the seed protein would be as connected to other seed proteins (directly or indirectly) by chance versus what is observed. Genes with the lowest p-values are highlighted in red as shown in the color bar. The grey nodes are the common interactors with the seed proteins.

Reference

1. Kang HJ, Kawasawa YI, Cheng F, Zhu Y, Xu X, Li M, Sousa AM, Pletikos M, Meyer KA, Sedmak G, et al: **Spatio-temporal transcriptome of the human brain.** *Nature* 2011, **478:**483-489.
2. Stein JL, Torre-Ubieta Ldl, Tian Y, Parikshak NN, Baker DK, Lu D, Lowe JK, Wexler EM, Geschwind DH: **A Quantitative Framework to Evaluate Modeling of Cortical Development by Neural Stem Cells.** *Neuron* in press.
3. Benjamini Y, Hochberg Y: **Controlling the False Discovery Rate - a Practical and Powerful Approach to Multiple Testing.** *J Roy Stat Soc B Met* 1995, **57:**289-300.
4. Matys V, Fricke E, Geffers R, Gossling E, Haubrock M, Hehl R, Hornischer K, Karas D, Kel AE, Kel-Margoulis OV, et al: **TRANSFAC: transcriptional regulation, from patterns to profiles.** *Nucleic Acids Res* 2003, **31:**374-378.
5. Matys V, Kel-Margoulis OV, Fricke E, Liebich I, Land S, Barre-Dirrie A, Reuter I, Chekmenev D, Krull M, Hornischer K, et al: **TRANSFAC and its module TRANSCompel: transcriptional gene regulation in eukaryotes.** *Nucleic Acids Res* 2006, **34:**D108-110.
6. Rossin EJ, Lage K, Raychaudhuri S, Xavier RJ, Tatar D, Benita Y, Cotsapas C, Daly MJ: **Proteins encoded in genomic regions associated with immune-mediated disease physically interact and suggest underlying biology.** *PLoS Genet* 2011, **7:**e1001273.
